# The Status and Research Progress on Vitamin D Deficiency and Atrial Fibrillation

**DOI:** 10.21470/1678-9741-2018-0322

**Published:** 2019

**Authors:** Lizhan Bie

**Affiliations:** 1 Department of Cardiology, the Third People’s Hospital of Yancheng, Yancheng, China.

**Keywords:** Atrial Fibrillation, Vitamin D Deficiency, Pathophysiology, Epidemiology

## Abstract

Atrial fibrillation is a common type of arrhythmia and is an important cause of stroke and heart failure. vitamin D is an emerging risk factor of AF, and is implicated in the pathophysiology of atrial fibrillation. It has been established that this vitamin is extensively involved in the regulation of both the renin angiotensin aldosterone system and the immune system. Epidemiological studies have not yet reached a consensus on the possible association between vitamin D deficiency and atrial fibrillation. Better research designs and methods can further clarify the relationship between the two.

**Table:** 

Abbreviations, acronyms & symbols	
AF	= Atrial fibrillation
CABG	= Coronary artery bypass grafting
FGF-23	= Fibroblast growth factor 23
IL	= Interleukin
NA	= Not available
POAF	= Postoperative atrial fibrillation
PTH	= Parathyroid hormone
RAAS	= Renin-angiotensin-aldosterone system
TNF-α	= Tumor necrosis factor alpha
VDBP	= Vitamin D-binding protein
VDR	= Vitamin D receptor

## INTRODUCTION

Atrial fibrillation (AF) is the most common type of arrhythmia. It is not a fatal arrhythmia itself, but it can impair the quality of life and can easily lead to stroke and increase the risk of cardiac death. AF epidemiological studies have concluded that the prevalence of AF is underestimated, the most important reason is the missed diagnosis of paroxysmal AF and the low enthusiasm for patients with asymptomatic AF. As the age structure of the population changes, the proportion of elderly increases, and other risk factors such as hypertension, diabetes, and cardiovascular disease increase, it is foreseeable that the future prevalence of AF will continue to increase.

At present, the pathogenesis of AF is not clear. Although antiarrhythmic drugs are widely used in clinical treatment, the effects of these drugs are not satisfactory due to frequent recurrence of AF and significant proarrhythmic effects^[1]^. Radiofrequency ablation and other procedures for patients with AF are not ideal for long-term benefit; therefore, upstream treatment of AF, the prevention of AF episodes is more important. Exploring targets that are new and easy to detect and control has become a new goal for the treatment of AF. Since 1990, Kessel^[2]^ reported a case of taking vitamin D to cure chronic AF, and the relationship between vitamin D deficiency and AF has gradually become a research hotspot.

## POSSIBLE MECHANISM

In the past studies, vitamin D research had been concentrated on bone metabolism. In recent years, studies have found out that vitamin D deficiency is involved in the pathogenesis of hypertension, heart failure, coronary heart disease, and obesity^[3]^. These factors are also common risk factors for AF. The biological effects of vitamin D are mainly derived from its binding to the nuclear steroid hormone vitamin D receptor (VDR), which exists in almost all tissues, including cardiac myocytes, and is involved in the structural changes of cardiac cells^[4]^.

It is well known that AF is closely associated with an inflammatory milieu. Vitamin D can increase the production of interleukin (IL)-10 and reduce the production of IL-6, IL-12, interferon-γ, and tumor necrosis factor alpha (TNF-α), thereby generating a cytokine spectrum that is conducive to reduce inflammation^[5]^ and the reduction of vitamin D and vitamin D-binding protein (VDBP) as part of the acute inflammatory system response. However, studies have suggested that vitamin D deficiency may be a consequence, not a cause of inflammation^[6]^. And vitamin D supplementation may delay the inflammatory response caused by decreased VDR function^[7]^. Vitamin D is a negative endocrine regulator of the renin-angiotensin-aldosterone system (RAAS)^[8]^, and low plasma 25-hydroxyvitamin D3 levels may result in upregulation of the RAAS^[9]^, while inhibition of the RAAS can prevent the onset of AF^[10]^. Vitamin D deficiency may impair the prevention of AF by inhibiting RAAS^[11]^.

The study found out that hemodialysis patients may inhibit the expression of vitamin D through fibroblast growth factor 23 (FGF-23) and promote the formation of AF^[12]^. Mathew demonstrated a consistent association between high circulating FGF-23 concentrations and an increased risk of developing AF^[13]^. High FGF-23 levels inhibit 1-alpha hydroxylase activity and reduce vitamin D3 production^[14]^. It is confirmed from the side that vitamin D deficiency is associated with AF.

Hanafy et al.^[15]^ found out that vitamin D can extend the action potential duration without increasing the contraction pressure of the left atrium. It can also reduce AF attacks and even terminate AF. And it did not prolong QT interval and did not increase the chance of ventricular arrhythmia. Canpolat et al.^[16]^ found out that the electromechanical delay in AF patients with vitamin D deficiency was significantly increased, and the electromechanical delay of atrium after vitamin D supplementation was reduced. The incidence of AF was increased by the electromechanical delay of atrium. These studies suggest that vitamin D supplementation may be a potential treatment for AF.

## EPIDEMIOLOGICAL RESEARCH

The pathogenesis of AF itself is not clear, so it is more meaningful to explore the relationship between AF and vitamin D from an epidemiological perspective. Table 1 summarizes the recent studies on the relationship between vitamin D deficiency and AF.

**Table 1:** Epidemiological study of atrial fibrillation (AF) and vitamin D deficiency.

Study (year)	Subject	Total patients	Result	
			Prevalence	Incidence
Rienstra et al.^[17]^	Two American community cohorts without prevalent AF	2930	NA	No
Vacek et al.^[20]^	A cohort of patients	10899	Yes	NA
Vitezova et al.^[18]^	A community-based cohort of middle-aged and elderly participants	3395	NA	No
Alonso et al.^19]^	Four communities in the United States of America	12303	No	No
Shadvar et al.^[23]^	Patients undergoing CABG	50	NA	Yes
Emren et al.^[24]^	Patients undergoing CABG	283	NA	Yes
Cerit et al.^[21]^	Patients undergoing CABG	128	NA	No
Gode et al.^[25]^	Patients undergoing CABG	90	NA	Yes
Özsin et al.^[26]^	Patients undergoing CABG	100	NA	Yes
Belen et al.^[33]^	Patients with chronic heart failure	180	Yes	NA
Ozcan et al.^[32]^	Hypertensive patients	227	NA	Yes
Canpolat et al.^[29]^	Lone paroxysmal AF	96	Yes	NA
Demir et al.^[30]^	Successive cardiology outpatient	298	Yes	NA
Qayyum et al.^[27]^	Patients in the department of cardiology with AF	258	No	NA
Chen et al.^[31]^	Non-valvular persistent AF	322	Yes	NA
May et al.^[28]^	A general cardiovascular population without depression (≥ 50 years old)	7358	No	NA
Fusaro et al.^[12]^	Hemodialysis patients	314	Yes	NA

CABG=coronary artery bypass grafting; NA=not available

First, in a large sample of community studies, the relationship between AF and vitamin D deficiency appears confusing. Rienstra et al.^[17]^, Vitezova et al.^[18]^, and Alonso et al.^[19]^ found out that there were no clear relations, but Vacek et al.^[20]^ thought they were related. The biggest difference between the two types of studies was the age of the study population. The average age of the study population in Rienstra was 66 years, the age of those in Vitezova was 71 years, and the study population in Vacek was relatively young, with an average age of 55 years. Among the risk factors for the incidence of AF, age is an independent and relatively important one. With increasing age, the risk factors for AF are high. Alonso's study was interesting, he found out that vitamin D deficiency was not associated with AF risk in different genders and ethnic groups, and as with other prospective studies, meta-analysis did not support the correlation between these two. However, he also pointed out that there was still a correlation between AF and vitamin D deficiency in a relatively young population (< 58). The lower the concentration of vitamin D, the more likely it was to contribute to the formation of AF, while in the old population, the effect was not obvious.

Secondly, in the classification study with relatively small sample size, the relationship between AF and vitamin D deficiency in different types of patients is also widely debated.

The relationship between vitamin D deficiency and postoperative AF (POAF) in patients is also unclear. Cerit found out that although there was a significant negative correlation between vitamin D and left atrial diameter, it was surprising that regression analysis showed that vitamin D deficiency was not an independent predictor of POAF^[21]^. In subsequent studies, he found out that vitamin D supplementation can prevent the occurrence of POAF when vitamin D is extremely deficient^[22]^. The other four studies supported that vitamin D deficiency directly led to an increased incidence of POAF^[23-26]^. The patients enrolled in the study by Gode et al.^[25]^ and Özsin et al.^[26]^ were young, and all studies supported a left atrial diameter as an independent risk factor for POAF. Overall evidence of vitamin D deficiency seems to support the onset of POAF.

In studies about the type of AF and the degree of vitamin D deficiency, Qayyum et al.^[27]^ found out that the degree of vitamin D deficiency was not associated with the type of AF episode and it was not associated with a thrombotic event. May et al.^[28]^ conducted a stratified analysis of the concentration of vitamin D in cardiovascular patients over 50 years of age, and found out that the prevalence of AF was basically the same under different levels of vitamin D deficiency. However, the subjects of this type of study were also older and had more chronic diseases than the ones of other studies.

The results of studies on non-valvular AF, especially lone AF and vitamin D deficiency, are encouraging. Canpolat et.al.^[29]^ found out that vitamin D deficiency and progression of AF were associated in a controlled study of patients with lone paroxysmal AF and healthy people, and that atrial fibrosis was demonstrated in patients with vitamin D deficiency by magnetic resonance imaging (MRI); more significantly, atrial fibrosis is an important marker of AF, and multivariable Cox regression analysis found out that vitamin D lacks were an independent risk factor for atrial fibrosis. Demir et al.^[30]^ found out that the level of vitamin D in patients with non-valvular AF was significantly lower than in patients without AF and with valvular AF, and that parathyroid hormone (PTH) levels were significantly higher in patients with non-valvular AF than in those without AF. This result suggested that hyperparathyroidism secondary to vitamin D deficiency may play a role in AF. The clinical effect of vitamin D deficiency was thought to be the result of reduced calcium absorption, which, in turn, increased PTH levels, but vitamin D levels in patients with valvular AF were similar to those in healthy controls. Chen et al.^[31]^ rigorously screened 162 patients with chronic AF (excluding many chronic diseases: diabetes, hypertension, coronary heart disease, etc.) and 160 healthy individuals without AF, and found low vitamin D levels associated with the prevalence of AF. Ozcan et al.^[32]^ also rigorously screened subjects and found out that vitamin D deficiency was associated with new-onset AF in hypertension. Also, in patients with chronic heart failure, low vitamin D concentrations were an independent risk factor for non-valvular AF, and the study also ruled out many chronic diseases^[33]^.

Through the meta-analysis of several studies, it was found out that vitamin D deficiency was associated with the occurrence of AF. In patients with chronic AF, vitamin D deficiency was associated with the development of AF, and the risk of AF increased to a certain extent^[34]^; while focusing on new-onset AF, there was no significant association between vitamin D status and risk of AF^[34,35]^; however, due to the heterogeneity of the studies included in this study, the results were not convincing.

## CONCLUSION

**Fig. 1:**
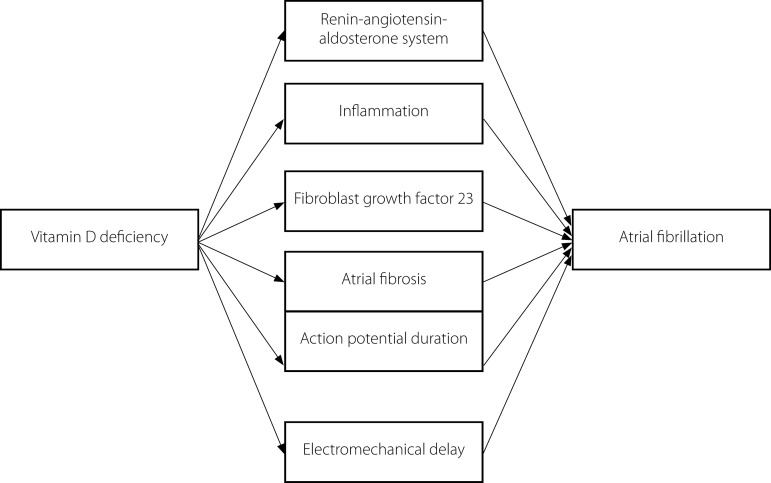
Mechanism of vitamin D deficiency leading to atrial fibrillation.

The relationship between vitamin D deficiency and AF still requires more basic research support in the study of mechanisms (Figure 1), and the results of epidemiological investigations are more convenient for guiding clinical work. Through several research findings, it can be inferred that vitamin D is in the process of AF, and the weight is not large compared with other risk factors, such as advanced age, hypertension, and coronary heart disease. The connection between the two is not suitable for large-scale community survey research and is not suitable for research in the elderly and people with more basic diseases. The exploration of the relationship between the two requires a more accurate classification of research. There may be unexpected surprises in the study of designated populations, such as patients with isolated AF and patients with low-grade non-valvular AF. Studies on whether vitamin D supplements prevent AF also need to be precisely classified.

**Table:** 

Authors' roles & responsibilities	
LB	Agreement to be accountable for all aspects of the work in ensuring that questions related to the accuracy or integrity of any part of the work are appropriately investigated and resolved; final approval of the version to be published.
